# Supplementary material for: Bull efficiency using dairy genetic traits
Source: PLoS One. 2019 Nov 11;14(11):e0223436. doi: 10.1371/journal.pone.0223436 (PMC6844452; doi:10.1371/journal.pone.0223436)
Supplement: Attachment — Submitted filename: Response to Reviewers.docx [file pone.0223436.s001.docx]

Reviewer #1:

**1. Manuscript is technically sound and data supports the conclusions. I answered 'partly' to this question because greater rigor would have looked at the effects over time. The current study is one time point and additional time points of bull data could have strengthen the generality of conclusions. Also, a linear combination of bulls could be appropriate if one evaluated a herd of cows. The Free Disposal Hull approach would not be needed for bull selection in a herd.**

We agree that analysis over time with updated bull lists would be an interesting application. In our summary, we state that analysis with an updated bull list could result in current bulls experiencing a decrease in calculated efficiencies as new bulls are added to the list. That should be expected with genetic progress. We leave that analysis for another paper.

Models that investigate bull selection given the current traits in the herd with the goal of increasing certain traits could utilize linear programming to maximize the value of the herd. Our analysis simple looks at the traits of a single bull with a comparison to every other bull, thus the use of the Free Disposal Hull technique.

**2. My criticism here is not of the statistical analysis, but of the reporting of the results. Four decimals is more than can be supported based on the standard deviations or sample size of approximately 400 bulls.**

We have made the recommended change. Now we are reporting our results with only two decimals instead of four.

**3. Data is available.**

The data is publicly available like you mentioned

**4. Most of the manuscript is presented well. A few sentences are missing words. Search for 'form' and replace with 'farm' in at least one place. 'Since' should often be replaced with 'Because'. Since implies time, which is not how it was used in most places. In many of the sentences with 'there are or there were', a revision of the sentences would improve readability.**

We searched the manuscript and corrected the words, ‘form to ‘farm’, and changed ‘since to because’ as you recommend and did a through read of the paper and made a few other editing changes to improve the readability of the manuscript.

5. **If my name will be associated with this review, I will advertise that I analyzed a similar data set with linear programing a few decades ago. (Shanks, R. D. and A. E. Freeman. 1979. Choosing progeny-tested Holstein sires that meet genetic goals at minimum semen costs. J. Dairy Sci. 62:1429-1434.) The market does a good job of arriving at a reasonable semen price for most bulls. When discrepancies occur, often some information not included in the data set is responsible for the outliers.**

Thank you for your suggestion. We added a sentence in the text citing the paper.
