# Supplementary material for: Bull efficiency using dairy genetic traits
Source: PLoS One. 2019 Nov 11;14(11):e0223436. doi: 10.1371/journal.pone.0223436 (PMC6844452; doi:10.1371/journal.pone.0223436)
Supplement: Attachment — Submitted filename: Response to Reviewers.docx [file pone.0223436.s002.docx]

You suggest the title, “Bull Efficiency of Dairy Genetic Traits” which is better than our title. However, we think that placing the word “bull” before the word “genetic” rather than “efficiency” is more correct and informative. Thus our new title is “Efficiency of Dairy Bull Genetic Traits”

line 69, lines 106 to 107, lines 172 to 173, line 359: How many times does Free Disposal Hull need to be defined as FDH? Unfortunately, I did not look up a PLOS ONE policy for abbreviations. Also, can an abbreviation start a sentence as in line 69?

I looked up the PLOS ONE policy for abbreviations and it states “Define abbreviations upon first appearance in the text.” I have edited the manuscript to fit the correct guidelines.

line 72: Consider replacing "efficiency" with "efficient" in this context.

Replacement was made.

line 116: "predicted transmitting abilities (PTA)

Correction made.

line 117 to 118: "genomic predicted transmitting abilities (GPTA)

Corrections made.

line 196: Is n the number of traits in this application?

I added the wording, “where *i*=1,…, n is the number of traits:” before equation one to address this comment.

line 301 to 303: Profit efficiency looks to be slightly to the right of revenue efficiency distribution to me on Figure 4. Are labels correct?

The labels are correct and I fixed my mistype from “left” to “right”. Thank you for finding this error.

line 348: Delete extra "to need".

Deleted as suggested.

lines 376 to 380: Interesting interpretation. I am not suggesting any changes, but consider that value of traits may not be solely additive with future analyses.

I included an extra sentence stating your suggestion.

Figure 1: If possible include a few more definitions for this figure. What is P1/P2? Is line AF perpendicular to P1/P2 line? From figure 2, I surmise that line AF should go through zero, zero and not necessarily be perpendicular. My guess is that my questions are pretty basic for the method, but I did not take the time to find the FDH reference.

I included an extra sentence at lines 181-182 where to elaborate on figure 1.
